# New Trends in Prevention

**DOI:** 10.34763/jmotherandchild.20212503SI.edit.2021_25_03SI_2

**Published:** 2022-02-09

**Authors:** Dorota Kleszczewska

**Affiliations:** 1 Institute of Mother and Child Foundation, Warsaw, Poland

## New trends in prevention

Looking at the development of prevention and educational programs targeting school-age children and adolescents, two issues are worth commenting on more broadly: concern for the high quality and effectiveness of the implemented programs and ensuring a variety of funding sources and the sustainability of such funding.

These issues are, in my opinion, the most serious challenges for people in charge, such as researchers, decision-makers and experts involved in the subject of adolescents’ public health. Why? First of all, young generations are the most difficult target groups. Who knows how to effectively communicate with them? What do they like, and will they get engaged in a recommended educational program?^1^ In answer to those concerns, we see a new trend in international research which has recently arisen^2^. It is called the *youth engagement approach*. The element of adolescents’ participation could be present at every step of the research process: from constructing the questionnaire, collecting the data, through comment on results, to the dissemination of the effects of the study^3^. In this special issue authors present the effects of this form of research in the context of the determinates of adolescents’ mental health problems (Kleszczewska et al.). Interesting conclusions on the matter of natural willingness of Polish adolescents to be engaged in prosocial activities drives Porwit et al. The authors emphasize that when planning activities aimed at involvement of young people, it is worth considering the specificity of various demographic and social groups.

The other solution on how to effectively reach young people with health-promoting programmes could be an alliance between organizations, and the exchange of international knowledge and best practices between experts from various research centers. This attitude is presented in the papers published in this issue, where international elements and synergy in research on adolescents’ public health is crucial^4^. Dzielska in her paper describes the validation of the active transport to and from school scales, which have been used in the ACTS project, in which research centers from Germany, Portugal, Netherlands, Poland and the Czech Republic joined their forces to promote physical activity of young people. The international knowledge exchanges and research results are presented also by Gajda et al.

The second challenge mentioned is the sustainability and effectiveness of public health programmes; gaining funding very often means meeting these criteria. The funding issue is strongly raised in the paper by Kamiński et al., in which practical directions are set. The aim of this paper was to identify the factors of success in medical crowdfunding campaigns. This paper could be very interesting for all those involved in fundraising for medical projects. I assume that these authors’ conclusions and remarks could also be useful for public health intervention organisers.

The quality of prophylactics addressed to children and young people in Poland as well as in many other countries leaves much to be desired. It is common practice to use programs that have no scientific basis, have not been evaluated, and as a result are ineffective. Schools continue to use ineffective approaches limited to imparting knowledge and communicating risks. Alternatives include active workshop work with students and strengthening personal resources that promote health. The classification of 40 basic resources (assets – health assets) supporting proper development is described in article by Porwit et al., that incorporates also a simpler division into external and internal factors divided into four main categories.

In this connection a System of Prophylactic Programs’ Recommendation and Promotion of Mental Health has been developed and implemented in Poland during the years 2006–2010. The system is operating and is being expanded. Its main purpose is to assess programs related to mental health promotion and prevention of risky behaviours, including use of psychoactive substances. Programs which fulfil quality criteria receive recommendations and information about the programs themselves and the principles of their propagation are included in a publicly available database (www.programyrekomendowane.pl)^5^. The aim of the system, apart from creating a bank of verified programs, is to popularise knowledge about effective prophylactics and promote good programs on a national scale. Schools can then resort to proven models, while teachers can benefit from assistance in the form of training and didactic materials.

A program may receive recommendation at one of the following three levels^1^:

1. A promising program: a well-structured program with process evaluation results justifying the assumption that the expected results of implementation will be achieved.
2. Good practice: a well-structured program, its effectiveness confirmed in evaluation tests with regard to its impact on factors which lead to achieving behaviour changes, in other words confirmed effectiveness with regard to the detailed objectives of the program.
3. Model program: a program with effectiveness confirmed in evaluation tests with regard to impact on problem behaviour.

The database of recommended programs also contains Polish adaptations of programs originating from other countries.

I believe that this issue prepared by authors from various organisations – universities, NGOs, research institutes, and with international guest editor’s involvement – will be a valuable resource for all interested in effective and sustainable prevention-based health programmes. The wide-ranging scope of public health matters presented in this issue offers efficient and useful directions for supporting young people in the problems of their daily lives.

Dorota Kleszczewska, PhD

Guest Editor

Institute of Mother and Child Foundation
